# Waist circumference does not predict circulating adiponectin levels in sub-Saharan women

**DOI:** 10.1186/1475-2840-6-31

**Published:** 2007-10-16

**Authors:** Eugène Sobngwi, Valery Effoe, Philippe Boudou, Dieudonné Njamen, Jean-François Gautier, Jean-Claude Mbanya

**Affiliations:** 1 Department of Medicine and Specialties, Faculty of Medicine and Biomedical Sciences, University of Yaoundé 1, Yaoundé, Cameroon; 2 Institute of Health and Society, Newcastle University, Newcastle upon Tyne, UK; 3 Department of Diabetes and Endocrinology, Saint-Louis University Hospital and INSERM U671, Paris, France

## Abstract

**Background:**

Because of previously reported ethnic differences in determinants and markers of obesity and related metabolic disorders, we sought to investigate circulating levels of adiponectin and their correlates in a sub-Saharan African (sSA) population.

**Subjects and Methods:**

We studied 70 non-diabetic volunteers (33M/37F) living in Yaoundé, Cameroon, aged 24–69 yr, with BMI 20–42 kg/m^2^. In all participants we measured waist circumference and total body fat by bioimpedance, and obtained a fasting venous blood sample for measurement of plasma glucose, serum insulin and adiponectin concentrations. We performed a euglycaemic hyperinsulinaemic clamp in 1/4 subjects, and HOMA_IR _was used as surrogate of fasting insulin sensitivity index since it best correlates to clamp measurements.

**Results:**

Males had lower adiponectin levels than females (8.8 ± 4.3 vs. 11.8 ± 5.5 μg/L). There was no significant correlation between adiponectin and total body fat (r_s _= -0.03; NS), whereas adiponectin was inversely correlated with waist circumference (r_s _= -0.39; p = 0.001). Adiponectin correlated negatively with insulin resistance (r_s _= -0.35; p = 0.01). In a regression analysis using fasting adiponectin concentration as the dependent variable, and age, HOMA_IR_, waist circumference, and fat mass as predictors, waist circumference (β = -3.30; p = 0.002), fat mass (β = -2.68; p = 0.01), and insulin resistance (β = -2.38; p = 0.02) but not age (β = 1.11; p = 0.27) were independent predictors of adiponectin. When considering gender, these relations persisted with the exception of waist circumference in females.

**Conclusion:**

Adiponectin correlates in this study population are comparable to those observed in Caucasians with the exception of waist circumference in women. The metabolic significance of waist circumference is therefore questioned in sSA women.

## Introduction

Adiponectin, an adipose-specific protein, has gained much attention due to its effect on glucose homeostasis and regulation of energy metabolism, making it a candidate link between obesity and insulin resistance [1], and a potential therapeutic pathway. Studies in Caucasians indicate that circulating adiponectin levels are lower in patients with type 2 diabetes, obesity, and cardiovascular disease than in background populations [2]. Circulating adiponectin levels correlate inversely with body mass status and positively with insulin sensitivity, measured either by the hyperinsulinaemic euglycaemic clamp technique or other surrogate methods [3-6]. However, there are indications from metabolic in vivo and in vitro studies that ethnic differences exists in the way adipose tissue and its distribution influences whole body metabolism. For example there are reports of lower antilipolytic effect of insulin in women of African descent compared to Caucasians [7,8]. Moreover, the relation between insulin sensitivity and the topographic distribution of adiposity is subject to ethnic-specific variations [9], but circulating levels and correlates of adiponectin in sub-Saharan Africans have not been investigated. In the present study, we examined, in healthy non-diabetic volunteers, the associations between circulating serum adiponectin concentrations and insulin sensitivity measured by a surrogate insulin sensitivity index, validated against the hyperinsulinaemic euglycaemic clamp technique.

## Methods

We studied 70 non diabetic Cameroonians (33 males and 37 females) aged 42 ± 10.6 years. All participants had a stable weight (< 5% variation) over 3 months prior to the study. Individuals were excluded from the study if they had physician – diagnosed diabetes, fasting blood glucose concentration > 1.25 g/L, any ongoing disease except well-controlled hypertension, or if they were currently receiving medications known to affect glucose and/or lipid metabolism. We performed all measurements at the National Obesity Centre, Yaoundé, starting between 8 and 10 AM after a 10 to 12-hour overnight fast. Participants were advised to avoid intense physical activity the day prior to, and on the day of the investigation and to have their last meal comprising 50–55% carbohydrates not later than 8 PM the day preceding investigations.

### Anthropometric measurements

Weight was measured to the nearest 0.1 kg (SECA, Germany). Height was measured to the nearest 0.5 cm using a wall – mounted stadiometer. BMI was calculated as weight (in kg) divided by the square of the height (in metres). Waist circumference was measured to nearest 1 cm at the level of the umbilicus (L_4 _- L_5_) and at the end of expiration with the subject upright and his/her hands by the side. Percentage body fat and total fat mass were measured by bioelectric impedance analysis (OMRON BF 302, OMRON Matsusaka Co., Ltd. Japan). For each parameter, 2 measurements were taken and the average used in the analysis.

### Metabolic investigations

In all the subjects we collected two fasting venous blood samples from an antecubital vein for the determination of circulating glucose, insulin and adiponectin levels. In a representative sub sample of 16 subjects, we performed a 120-min euglycaemic hyperinsulinaemic clamp at 80 mU/min/m^2 ^insulin infusion rate in order to measure whole body insulin sensitivity. Fasting insulin sensitivity indices were derived from fasting insulin and glucose measurements and were compared to the clamp measurements and yielded the following correlation coefficients: Fasting insulin (r = -0.70; p = 0.008), HOMA_IR _(r = -0.76; p = 0.004) and QUICKI (r = 0.67; p = 0.01) Glucose/Insulin (r = 0.50; p = 0.06). HOMA had the highest correlation coefficient with clamp-measured whole body insulin sensitivity and was therefore used as surrogate of insulin sensitivity in the study population.

### Biochemical measurements

Plasma glucose concentration was determined using the glucose oxidase method. Adiponectin and insulin levels (intra-assay CV = 1.78–6.21%) were determined by RIA (Linco Research Inc, St Charles, MO, USA) at the St-Louis University Hospital, Department of Hormonal Biology, Paris. Adiponectin and insulin were measured on serum samples that had been stored at -80°C for 2–4 months and shipped in thermo boxes on dry ice. All assays were run in duplicate.

### Statistical analyses

Statistical analyses were performed using the Statistical Package for Social Sciences, SPSS^® ^for Windows, Version 12. Results are expressed as frequencies or mean ± SD unless otherwise stated. Comparison across groups was done using one-way analysis of variance (ANOVA) with further group-to-group comparison using the non-parametric Mann-Whitney U test. Correlations between variables was analysed using the non-parametric Spearman Rank Order Test. Linear regression analysis was used to determine independent predictors of serum adiponectin levels.

### Ethical considerations

The study protocol was approved by the Review Committee of the Faculty of Medicine and Biomedical Sciences, University of Yaoundé 1, Cameroon. The study was conducted according to the principles expressed in the Declaration of Helsinki, and written informed consent was obtained from all participants.

## Results

### Characteristics of the study population

The study population comprised 33 males aged 41.7 ± 10.5 years, and 37 women aged 42.2 ± 10.9 years (NS). Of the 70 subjects, 16 (9M, 7F) had a BMI < 25 kg/m2 (normal weight), 28 (17M, 11 F) had a BMI between 25 and 29.9 kg/m2 (overweight) and 26 (7M, 19F) had a BMI ≥ 30 kg/m2 (obese). Women had significantly higher total body fat compared to men (31.8 ± 9.2 vs. 20.7 ± 8.7%). Mean waist girth in cm was 93.0 ± 12.4 in women and 91.9 ± 11.1 in men (NS). Insulin sensitivity was also comparable in women and men (HOMA = 0.99 ± 0.51 vs. 1.02 ± 0.51, NS). Table 1 shows the characteristics of the study population by gender and BMI class.

**Table 1:** Characteristics of the study population by sex and obesity class

	**Normal weight (n = 16)**			**Overweight (n = 28)**			**Obese (n = 26)**		
			
	**M**	**F**	**P**	**M**	**F**	**P**	**M**	**F**	**P**
			
**N**	9	7		17	11		7	19	
**Age (years)**	37.1 ± 13.9	39.3 ± 7.5	0.72	41.4 ± 9.2	37.8 ± 10.9	0.36	48.1 ± 4.1	45.9 ± 11.0	0.61
**BMI (kg.m^**-2**^)**	22.2 ± 1.2	23.5 ± 1.4	0.07	27.5 ± 1.4	28.2 ± 1.4	0.20	32.2 ± 2.0	35.2 ± 3.1	0.03
**Fat mass (kg)**	12.2 ± 4.1	19.7 ± 3.8	0.002	20.5 ± 2.9	28.4 ± 2.5	0.000	32.0 ± 8.3	38.4 ± 7.0	0.06
**Waist girth (cm)**	77.3 ± 5.3	79.6 ± 3.2	0.35	95.4 ± 4.5	88.6 ± 6.1	0.005	103.7 ± 2.9	100.7 ± 11.5	0.51
**HOMA_**IR **_index**	0.68 ± 0.14	0.72 ± 0.34	0.93	0.96 ± 0.51	0.94 ± 0.42	0.91	1.36 ± 0.53	1.13 ± 0.61	0.41
**Adiponectin (mg/L)**	12.8 ± 3.4	13.0 ± 8.2	0.82	8.1 ± 4.0	11.6 ± 3.3	0.03	5.3 ± 1.7	10.7 ± 4.3	0.003

### Correlates of circulating adiponectin levels

Women had higher circulating adiponectin levels compared to men (11.4 ± 4.8 vs. 8.8 ± 4.3 mg/L, p = 0.02). As shown in table 1, adiponectin levels were higher in overweight and obese women compared to men, but not in the normal weight sub group in which the levels were comparable in women and men. Fasting adiponectin levels were highest in the normal weight group, when compared to the overweight and obese groups, but there was no significant correlation between adiponectin and BMI (*r*_*s *_= -0.16; p = 0.20) or with total body fat (*r*_*s *_= -0.03; p = 0.81). Adiponectin levels were inversely correlated negatively with waist circumference (*r*_*s *_= -0.39; p = 0.001) and insulin resistance as measured by the HOMA_IR_(*r*_*s *_= -0.353; p = 0.01).

### Gender-specific and multivariate analyses

**Figure 1:**
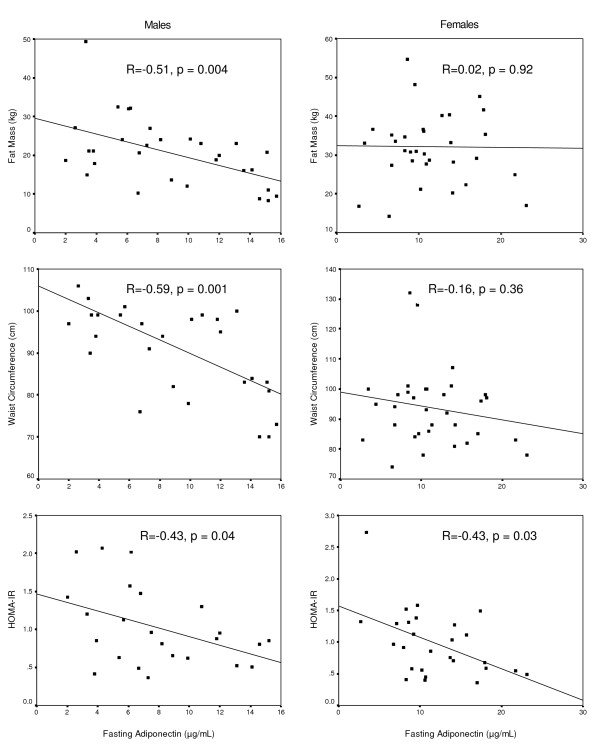
**Correlates of serum adiponectin levels by sex**. Bivariate correlation between fasting adiponectin levels and fat mass (upper panel), waist circumference (mid panel) and insulin resistance (lower panel) in men (left) and women (right). Spearman correlation coefficient (r) and significance (p) indicated in the figures.

In a regression analysis using fasting adiponectin concentration as the dependent variable, and age, HOMA_IR_, waist circumference, and fat mass as predictors, waist circumference (β = -3.30; p = 0.002), fat mass (β = -2.68; p = 0.01), and insulin resistance (β = -2.38; p = 0.02) but not age (β = 1.11; p = 0.27) were independent predictors of adiponectin. When taking into consideration gender, all the associations persisted in men. In women, the associations between adiponectin and total fat, and adiponectin and waist circumference were not statistically significant. Correlates of adiponectin in women and men are shown in figure 1.

## Discussion

The present study is the first investigation of adiponectin and its correlates in subjects from sub-Saharan Africa. It confirms most of the current knowledge on adiponectin acquired from other populations with the exception of a less clear relation with adiposity and the surrogates of its topographic distribution, especially in women. In fact, no correlation existed between adiponectin levels and waist circumference in sSA women in the present study.

While the measure of insulin sensitivity has been validated in the study group against the gold standard measure which is the euglycaemic hyperinsulinaemic clamp, it can be questioned whether waist circumference is an appropriate surrogate of intra abdominal fat. In fact, Lovejoy et al. observed that, at similar levels of BMI and waist-to-hip ratio, African-American women had smaller visceral fat compared to Caucasian women [10]. In addition WHR was correlated with fasting insulin only in Caucasians [10]. Similarly, the group of Albu found a lower visceral/sub cutaneous tissue in obese black women compared to whites at comparable levels of WHR [11]. These discrepancies between imaging techniques for the measurement of visceral adipose tissue and clinical surrogates would explain at least partially the need for ethnic-specific cut points for waist circumference in the definition of risk and/or metabolic syndrome as attempted by IDF in 2005 [12].

In addition to this, the present study provides a strong indirect indication of the questionable metabolic significance of waist circumference in the study population. In fact, contrasting with the unclear relation between waist circumference and adiponectin levels, waist was associated with insulin resistance. Population-based epidemiological studies had suggested similar assumptions since waist circumference was not associated with fasting and 2 h blood glucose in Cameroon but was the most consistent component of the metabolic syndrome [13]. A growing hypothesis concerns the effect of the duration of exposure to obesity that might explain the differences in the relation with metabolic outcome as would indicate life course studies [14]. Finally, recently Sniderman et al. developed the concept of adipose tissue overflow in Asians, hypothesising that superficial subcutaneous adipose tissue compartment is larger in whites than in South Asians, with a risk of earlier exhaustion of the storage capacity of their superficial subcutaneous adipose tissue compartment resulting in earlier metabolic complications of upper body obesity at lower absolute masses of adipose tissue [15]. We cannot exclude a similar possibility in Africans.

In conclusion, correlates of adiponectin in the sSA population studied are similar to observations in Caucasians, with the exception of the relation with waist circumference in women suggesting either the inappropriateness of waist as clinical surrogate for abdominal fat in this population, or a different metabolic meaning of this parameter. Therefore, further studies aiming at validating better clinical surrogates for abdominal fat are warranted.
